# The relationship between teamwork and moral distress among NICU nurses

**DOI:** 10.1186/s12912-024-02437-3

**Published:** 2024-10-28

**Authors:** Zeinab Alipour, Monir Nobahar, Raheb Ghorbani, Elahe Jahan

**Affiliations:** 1 Student Research Committee, Semnan University of Medical Sciences, Semnan, Iran; 2 Nursing Care Research Center, Semnan University of Medical Sciences, Semnan, Iran; 3 Department of Nursing, Faculty of Nursing and Midwifery, Semnan University of Medical Sciences, Semnan, Iran; 4 Social Determinants of Health Research Center, Semnan University of Medical Sciences, Semnan, Iran; 5 Social Medicine Department, Faculty of Medicine, Semnan University of Medical Sciences, Semnan, Iran

**Keywords:** Teamwork, Moral distress, Neonatal intensive care unit, Nursing staff

## Abstract

**Background:**

In the demanding environment of the neonatal intensive care unit (NICU), quality nursing care hinges on effective teamwork and communication among nurses. However, this requirement for close cooperation can expose nurses to significant levels of moral distress. This study aims to explore the connection between the quality of teamwork and the experience of moral distress among NICU nurses.

**Methods:**

Employing a cross-sectional, multicenter descriptive correlational design, this study surveyed female NICU nurses across the cities of Khorramabad and Semnan. Census sampling was utilized over five months, from July to November 2023, resulting in the participation of 190 nurses. Tools for data collection included demographic questionnaires, the Team-STEPPS Teamwork Perception Questionnaire (T-TPQ), and the Moral Distress Scale-Revised (MDS-R) for nurses.

**Results:**

The findings revealed an average teamwork score of 3.73 ± 0.78, denoting an acceptable level, and an average moral distress score of 91.2 ± 56.7, indicating a low level. In multiple linear regression, marital status showed a direct positive correlation (β = 38.5, SE (β) = 9.3, *p* < 0.001), while the number of children (β = -14.6, SE (β) = 4.9, *p* = 0.003) and the teamwork score (β = -1.1, SE (β) = 0.12, *p* < 0.001) were inversely correlated with moral distress.

**Conclusion:**

The study’s results suggest that stronger teamwork among nurses correlates with reduced moral distress. Enhancing teamwork within NICUs could lead to policy development focused on the safety and quality of newborn care, also potentially alleviating moral distress experienced by nurses.

## Background

Every year, fifteen million premature babies are brought into the world [1]; the prevalence of premature delivery and low birth weight in Iran, based on 62 studies with a total sample size of 301,839 babies, was found to be 7.95% (95% confidence interval [CI]: 7.36–8.58) [2]. Over the past two decades, the incidence of preterm births has increased, resulting in a higher number of admissions to the neonatal intensive care unit (NICU) [3]. As these infants are at a crucial phase for survival, it is concerning that 45% of deaths among children under five are infants [4]. NICUs serve a purpose beyond merely sustaining life [5]. The increasing complexity of neonatal care, challenging scenarios [6] such as end-of-life decisions, resuscitation, intricate technology use, high workloads, infant vulnerability, uncertain treatment outcomes, and sometimes non-beneficial medical interventions [7], all demand cohesive teamwork characterized by effective collaboration and communication [8].

Teamwork in healthcare is a dynamic, synergistic process among professionals with diverse but complementary skills working together towards shared health goals. Interprofessional teamwork is defined as a dynamic process involving two or more healthcare professionals with complementary backgrounds and skills, sharing common health goals and exercising concerted physical and mental effort in assessing, planning, or evaluating patient care [9]. Effective teamwork within health care not only ensures safer, high-quality nursing care but also fosters creativity, flexibility, and job satisfaction and reduces staff turnover [10]. Research by Marsh and Mc Nay indicates that teamwork substantially reduces patient morbidity and mortality [11].

Teamwork is essential to ensure the quality and safety of healthcare delivery in the intensive care unit (ICU) [12]. To provide quality care for infants and families, cohesive team dynamics are required, including professional competence, effective communication, accountability, collaboration, and mutual respect [13].

Advances in medical technology have allowed the smallest and sickest neonates to survive [14]. Moreover, NICU practices involving high-tech interventions, infant vulnerability, uncertain prognoses, and potential for both short-term and long-term treatment complications are correlated with significant moral distress [5, 15]. Defined as the anguish experienced when external factors constrain one’s professional actions despite knowing the morally proper course, moral distress arises when caregivers encounter barriers that prevent them from acting according to their ethical judgments [16, 17]. Specifically, when nurses’ moral obligations towards patients and their families are thwarted, leading to feelings of impotence and failure, they experience moral distress [18]. This distress is particularly pronounced among nurses, stemming from the intimate care they provide and the close relationships they form with infants and their families [19]. In challenging circumstances, where nurses perceive the care as not aligning with the infants’ best interests, they experience intense moral stress [15]. A study by Prentice et al. in Australia in 2017 indicated that 72% of NICU staff experience moral distress at least monthly [20], and a 2021 Tehran-based study by Tajalli et al. revealed that average moral distress levels among NICU nurses exceed 40% [5]. Yet, the overall prevalence of moral distress in NICUs remains unclear [21].

Moral distress can profoundly impact nurses both personally and professionally [22]. Individually, it may lead to depression, anxiety, anger, despair, physical ailments like headaches or sleep disorders [23–25], as well as a diminished sense of autonomy and feelings of helplessness [26]. Professionally, it can erode job satisfaction and result in subpar care quality, burnout, overall job dissatisfaction, and, eventually, job turnover [14].

Moral distress is one of the obstacles to achieving the goals of social systems, especially in healthcare, and it has been the focus of many researchers for more than three decades due to growing concerns about it [27]. The severity of the moral distress experience increases with the specialization of nursing duties and the increase in their responsibility to care for critically ill patients [28]. Additionally, evidence shows that poor communication and a lack of cooperation with other healthcare professionals in caring for critically ill patients are among the main reasons for moral distress among ICU nurses [29–32].

The limited ability to predict outcomes and uncertainty add to the challenges of moral distress. Differences in opinions and approaches among medical team members can strain communication and affect each person differently [14]. The results of Murphy et al.‘s (2018) study showed that teamwork and communication training measurably improved the safety attitudes of NICU staff [33]. Similarly, the study by Masten et al. (2019) indicated that aspects of providing team care in the NICU require further research [34]. Despite the high importance of teamwork and moral distress, particularly in the NICU setting where nursing care for vulnerable groups is critical, there is a notable lack of valid sources and literature addressing these issues among NICU nurses. Given the challenging nature of NICU work environments, which can affect neonates’ health and safety, and the significant impact that both inadequate teamwork and resultant moral distress may have on NICU nurses’ professional performance, it is critical to investigate these dynamics within an Iranian context where cohesive research is lacking. Therefore, this study aims to ascertain the relationship between teamwork and moral distress among NICU nurses.

## Methods

### Study design

This study employed a descriptive correlational design encompassing multiple centers. Census sampling was utilized over five months, from July to November 2023.

### Sample size

In this study, data were collected from 100 participants, and the correlation coefficient between teamwork scores and moral distress was found to be -0.402. Based on the results of this pilot study, with a 99% confidence level and 99% power, and considering an effect size factor of 1.5,

$$\:n=\frac{{\left({Z}_{1-\alpha\:}+{Z}_{1-\beta\:}\right)}^{2}}{{w}_{r}^{2}}+3$$

$$\:{w}_{r}=\frac{1}{2}Ln\frac{1+r}{1-r}$$

The required sample size was calculated to be 184 participants. Consequently, the final sample size was rounded up to 190 participants.

### Participants

The study focused on all female nurses working in the NICUs (*n* = 190) of 7 hospitals located in Khorramabad and Semnan cities. The capacity of these NICUs varies by hospital, with the number of beds ranging from 7 to 22. The sample consisted of 214 nurses, of whom 190 completed the questionnaire. The remaining 24 either did not meet the conditions to participate in the study, were on maternity leave, or did not fully complete the questionnaire.

### Inclusion criteria

Eligible participants included those with a bachelor’s degree in nursing (or higher) and a minimum of 6 months’ work experience in the NICU.

### Exclusion criteria

Nurses with physical or mental illness with self-report and those who submitted incomplete or distorted questionnaires were excluded from the study.

### Data collection instrument

Data were gathered using demographic information questionnaires, the Team-STEPPS^®^ Teamwork Perception Questionnaire (T-TPQ), and the Moral Distress Scale-Revised (MDS-R) for nurses.

### Demographic questionnaire

The demographic questionnaire collected information on age, gender, marital status, number of children, education level, work experience, employment status, income, organizational position, and shift type.

### Team-STEPPS teamwork perception questionnaire (T-TPQ)

The T-TPQ is an evidence-based tool to measure an individual’s attitude and perception of teamwork knowledge, skills, and behaviors. This tool assesses teamwork with 35 items across 5 subscales, Each subscale had 7 items: “team structure”, “team leadership”, “situational assessment”, “mutual support”, and “communication”. Each item is scored using a five-point Likert scale, ranging from strongly disagree (1) to strongly agree (5). An average score of 3 or higher is indicative of acceptable teamwork [35, 36]. The Persian version of this tool has demonstrated acceptable structural validity and internal reliability, with a reported Cronbach’s alpha coefficient of 0.96 [37].

### The moral distress scale-revised (MDS-R)

The Moral Distress Scale-Revised (MDS-R) is a tool for quantifying moral distress among nurses. Initially developed by Corley et al. in 2001 as a 38-item questionnaire [38], it was later refined by Hamrick, Burchers, and Epstein in 2012 into a version with 21 items for nurses [39]. In Iran, Otaghi oversaw the Persian translation and validation of the scale by Arab and Barzegari in 2014, which resulted in the reduction of the scale to 18 items [40]. The questionnaire utilizes a 5-point Likert scale to assess moral distress across two dimensions: frequency and intensity. Item scores range from “0 (never)” to “4 (very much)” in terms of frequency and from not at all (0) to very much (4) in terms of intensity, with a combined possible score of 0 to 16 per item. The overall moral distress score is determined by summing all item scores and can range from 0 to 288, where a higher score denotes greater moral distress. Scores are classified into three levels: “low (0–96)”, “moderate (97–192)”, and “high (193–288)” [5, 40]. The validity and reliability of the Persian version of this scale were established by Otaghi, Arab, and Barzegari in 2014, with experts affirming its content and external validity, and a reported Cronbach’s alpha of 0.75, suggesting satisfactory reliability [40]. Soleimani et al. (2019) also validated the scale, with an overall Cronbach’s alpha coefficient of 0.71 and sub-scale coefficients ranging from 0.853 to 0.685, indicating good reliability across the different components of the tool [41].

### Procedure

Before commencing data collection, necessary approvals were secured from the Ethics Committee of Semnan University of Medical Sciences and the Research Vice-Chancellor of Lorestan and Semnan Universities of Medical Sciences. All the nurses of the NICUs from 7 hospitals in Khorramabad and Semnan who met the entry criteria and were willing to participate in the study were selected. The nurses were briefed on the study’s nature, goals, eligibility criteria, and the confidentiality of their participation. Two researchers in charge of data collection approached the NICUs of 7 hospitals, public, private, and social security, across various shifts and weekdays, coinciding with periods of lower nurse workloads. They were stationed in these departments, and at appropriate times, when the nurses’ routine tasks were completed, and there was calm in the ward, they obtained written informed consent from the nurses. Following this, the questionnaires were provided to the nurses. Nurse participants were asked to complete the demographic, Teamwork: Team-STEPPS^®^, and moral distress questionnaires. The collected information was kept completely confidential and was not made available to anyone. To ensure ethical compliance, the nurses distributed the questionnaires anonymously, allowing them to complete them personally and return them to the researchers. The researchers considered the time needed to complete the questionnaires, which took an average of 20 to 30 min.

### Data analysis

The data were analyzed using Kolmogorov-Smirnov, Mann-Whitney, and Kruskal-Wallis tests, along with Pearson and Spearman correlation coefficients, and multiple linear regression. The SPSS24 software package was used for the analyses, with a predetermined significance level set at 0.05.

### Ethical considerations

The ethical considerations section addresses the measures taken to ensure the study abides by ethical research standards. The approval by the Semnan University of Medical Sciences Ethics Committee indicates that the study’s methodologies conformed to ethical guidelines. Preparations for data collection included obtaining official permissions from the respective hospital authorities. The purpose and methods of the research were communicated to the nurses, with an emphasis on confidentiality and the ethical management of personal information. Written informed consent was then obtained from the nurses, confirming their understanding and voluntary agreement to participate. The consent process suggests voluntary participation and concern for the autonomy and well-being of the nurses involved. Detailed information on the study objectives, confidentiality assurance, and the voluntary nature of participation demonstrate adherence to informed consent principles. Furthermore, the ethical code reference (IR.SEMUMS.REC.1402.059) implies that the specific ethical protocols and standards required by the local jurisdiction were met.

## Results

### Demographic information of the participants

All the nurses working in the NICUs of these hospitals were women. The mean ± standard deviation of the age of the nurses was 32.9 ± 5.8 years. The lowest age was 23, and the highest was 50 years. 69.5% (*n* = 132) were married, and the rest were single. 96.3% (*n* = 183) were bachelors, and the rest were masters. The mean ± standard deviation of the working experience of the nurses was 8.6 ± 5.7 years, and the lowest and highest working experience was 1 and 28 years, respectively. 92.6% (*n* = 176) were rotating shifts. 29.5% (*n* = 56) of the nurses were employed by plan or contract, and the rest were official or contracted. 47.9% (*n* = 91) of the nurses were in charge of the shift, and the rest were supervisors or just nurses. 26.8% (*n* = 51) of nurses had at least 2 children. The mean ± standard deviation of the nurses’ income was 13.0 ± 2.6 million tomans. 23.7% (*n* = 45) of nurses stated that their income is from 15 to 20 million tomans. The demographic characteristics of the participants are shown in Table 1.

**Table 1:** Distribution of individual characteristics of the examined nurses

Characteristics		*n*	%
Age (year)	< 30	60	31.6
	30–39	105	55.3
	≥ 40	25	13.2
Marital status	Single	58	30.5
	Married	132	69.5
Education	Bachelor	183	96.3
	Master	7	3.7
Work experience (year)	< 5	49	25.8
	5-9.9	67	35.3
	≥ 10	74	38.9
Shift work	Rotating shift	176	92.6
	Fixed shift	14	7.4
Type of employment	Contract	122	64.2
	Official	12	6.3
	Contractual	56	29.5
Job position	Head nurse	7	3.7
	Shift manager	91	47.9
	nurse	92	48.4
Number of children	0	78	41.1
	1	61	32.1
	≥ 2	51	26.8
Income (million tomans)	< 15	145	76.3
	≥ 15	45	23.7

### Outcome

Table 2 shows that the mean ± standard deviation of overall teamwork and moral distress was 3.73 ± 0.78 (out of 5) and 91.2 ± 56.7 (out of 288), respectively, and the average of all teamwork components was more than 3. Teamwork and moral distress ratings were reported using mean ± SD, median, and interquartile range (Table 2).

**Table 2:** Mean, standard deviation, median, and interquartile range of the scores of teamwork components and nurses’ moral distress

Parameter	Components	Score range	Mean	SD	Median	Interquartile range
Teamwork	Team structure	1–5	3.83	1.01	4.00	1.29
	Leadership	1–5	3.71	1.01	3.86	1.32
	Mutual Support	1–5	3.70	0.92	3.71	1.00
	Situational assessment	1–5	3.57	0.88	3.57	1.29
	Communication	1–5	3.86	0.78	4.00	0.89
	Overall Teamwork	1–5	3.73	0.78	3.89	0.91
Moral distress	Moral Distress	0-288	91.2	56.7	82.5	81

**Fig. 1:**
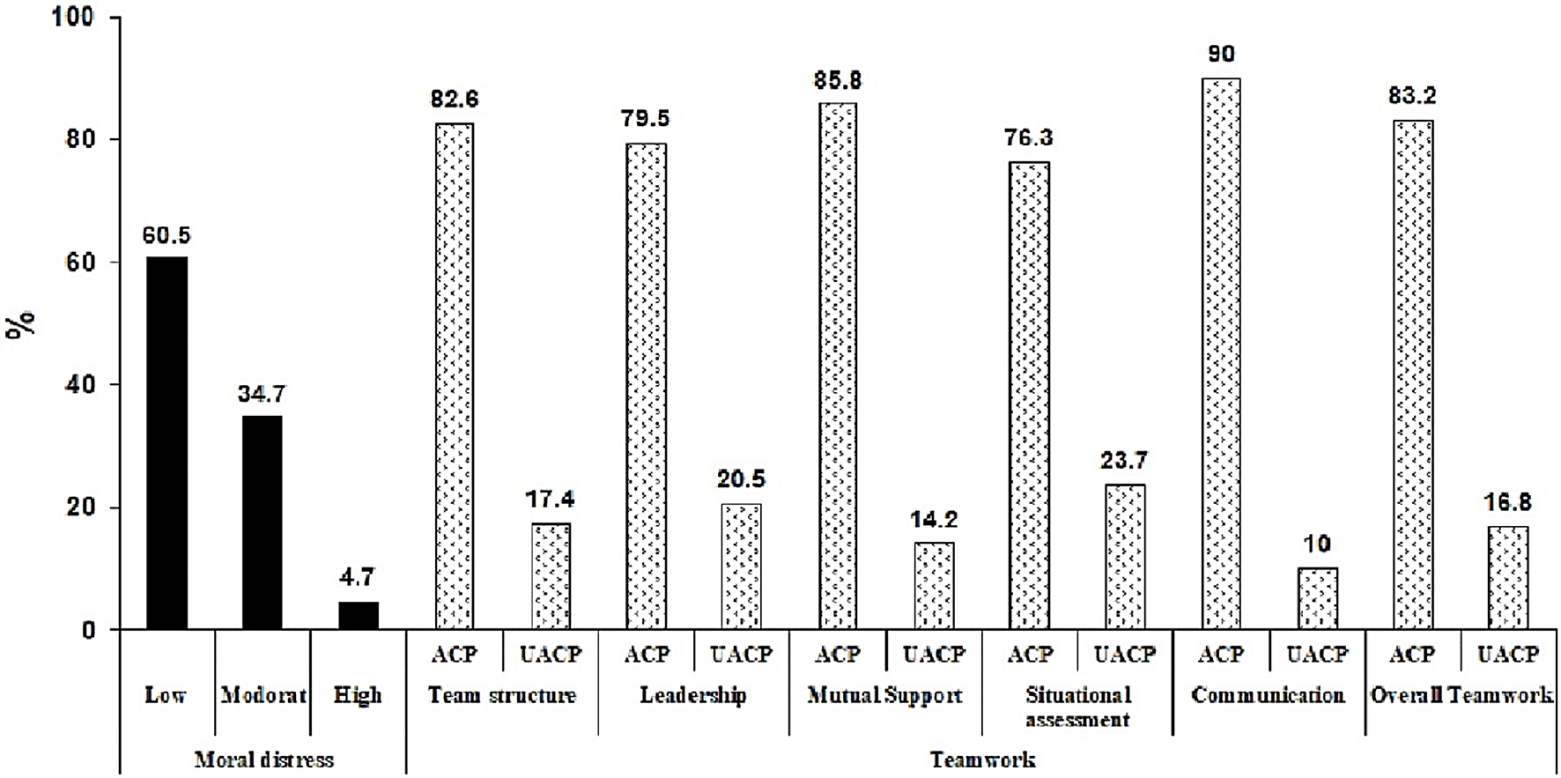
Distribution of teamwork and moral distress in nurses. ACP: acceptable UACP: unacceptable

Table 3, Fig. 1 shows that 83.2% of nurses had acceptable teamwork (≥ 3). More than 76% of nurses were acceptable in all teamwork components. The mean ± standard deviation of overall teamwork and moral distress were 3.73 ± 0.78 (out of 5) and 91.2 ± 56.7 (out of 288), respectively. The average of all teamwork components was more than 3. 60.5% of nurses had low moral distress, and 34.7% had moderate moral distress .

**Table 3:** Distribution of teamwork and moral distress in nurses

Parameter				*n*	%
Moral distress	0–96 (Low)			115	60.5
	97–192 (Moderate)			66	34.7
	193–288 (High)			9	4.7
Teamwork		Team structure	Acceptable (≤ 3) Unacceptable(< 3)	157	82.6
				33	17.4
		Leadership	Acceptable (≤ 3) Unacceptable(< 3)	151	79.5
				39	20.5
		Mutual Support	Acceptable (≤ 3) Unacceptable(< 3)	163	85.8
				27	14.2
		Situational assessment	Acceptable (≤ 3) Unacceptable(< 3)	145	76.3
				45	23.7
		Communication	Acceptable (≤ 3) Unacceptable(< 3)	170	90.0
				19	10.0
		Overall Teamwork	Acceptable (≤ 3) Unacceptable(< 3)	158	83.2
				32	16.8

**Fig. 2:**
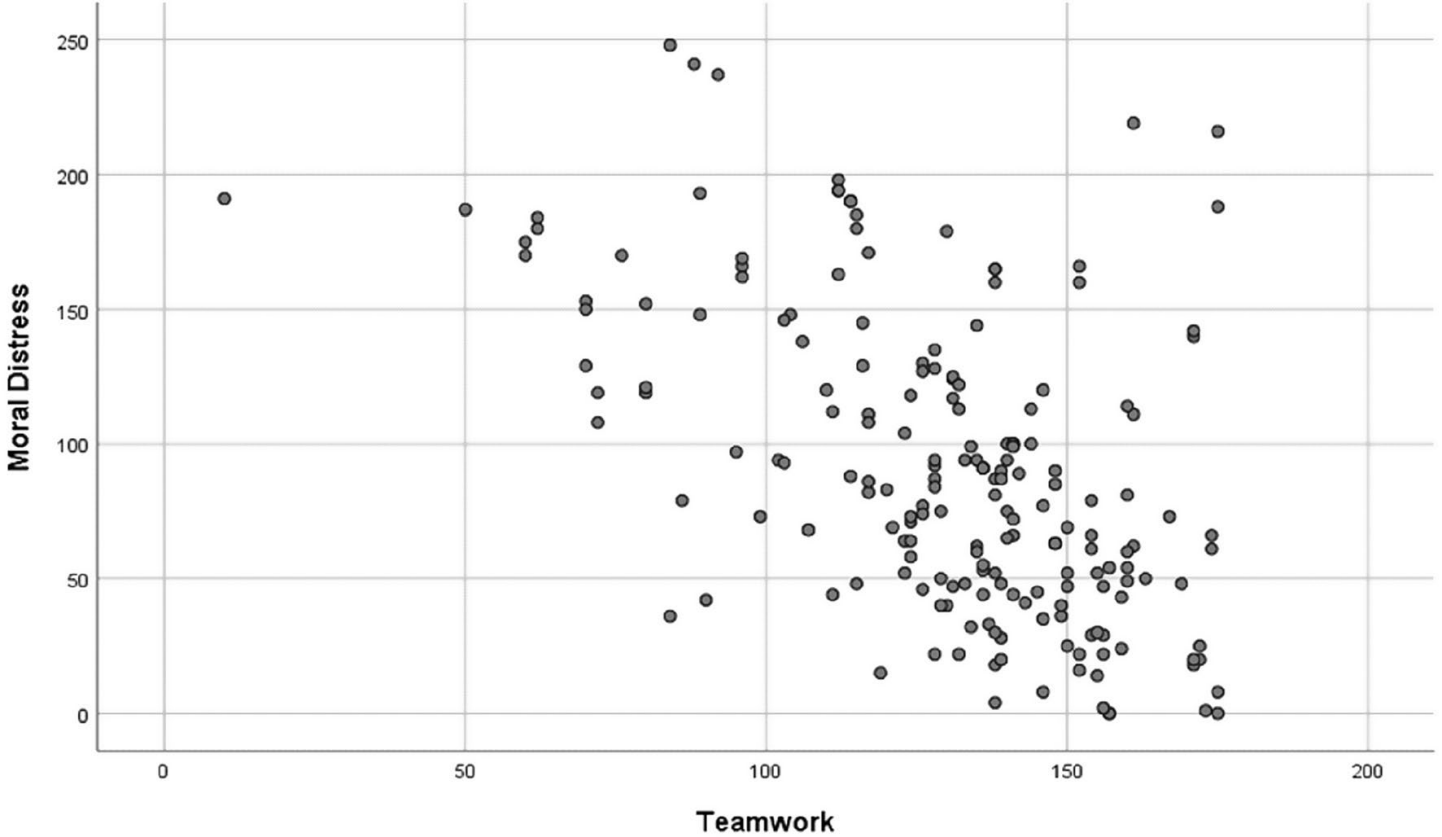
Scatter plot for correlation between Moral Distress and Teamwork

In the univariate analysis, teamwork had a significant inverse correlation with the moral distress score (*r* = -0.531 and *P* < 0.001). In the multiple linear regression analysis, being married had a direct correlation (β = 38.5, SE (β) = 9.3, *P* < 0.001), number of children (β=-14.6, SE (β) = 4.9, *P* = 0.003) and teamwork score (β=-1.1, SE (β) = 0.12, *P* < 0.001) showed an inverse correlation with moral distress score (Table 4, Fig. 2).

**Table 4:** Mean, standard deviation, and distribution of moral distress scores by individual characteristics of nurses

Specification	Characteristic	Moral distress								Correlation coefficient	P-Value
		Mean	SD	LOW		Moderate		High			
				*n*	%	*n*	%	*n*	%		
Age (year)	< 30	88.6	60.6	37	61.7	20	33.3	3	5.0	-0.043 ^b^	0.560
	30–39	98.7	56.4	56	53.3	44	41.9	5	4.8		
	≥ 40	66.1	39.9	22	88.0	2	8.0	1	4.0		
Marital status	Single	81.6	52.5	35	60.3	23	39.7	-	-	-	0.178 ^a^
	Married	95.5	58.5	80	60.6	43	32.6	9	6.8		
Education	Bachelor	91.1	57.0	110	60.1	64	35.0	9	4.9	0.022 ^c^	0.762
	Master	95.6	51.3	5	71.4	2	28.6	-	-		
Work experience (year)	< 5	82.2	51.4	32	65.3	16	32.7	1	2.0	0.039 ^b^	0.592
	5-9.9	96.7	64.5	37	55.2	27	40.3	3	4.5		
	≥ 10	92.2	52.4	46	62.2	23	31.1	5	6.8		
Shift work	Rotating shift	92.0	56.7	104	59.1	64	36.4	8	4.5	-	0.363 ^a^
	Fixed shift	81.9	58.1	11	78.6	2	14.3	1	7.1		
Type of employment	Contract	92.1	56.6	75	61.5	41	33.6	6	4.9	-	0.780 ^d^
	Official	75.9	33.7	8	66.7	4	33.3	-	-		
	Contractual	92.6	60.9	32	57.1	21	37.5	3	5.4		
Job position	Head nurse	56.3	20.1	60	65.2	28	30.4	4	4.3	0.060 ^c^	0.412
	Shift manager	100.6	59.3	48	52.7	38	41.8	5	5.5		
	nurse	84.6	54.3	7	100	-	-	-	-		
Number of children	0	92.3	56.4	44	56.4	32	41.0	2	2.6	-0.075 ^b^	0.306
	1	98.8	59.0	37	60.7	19	31.1	5	8.2		
	≥ 2	80.6	53.7	34	66.7	15	29.4	2	3.9		
Income (million tomans)	< 15	94.0	55.8	85	58.6	53	36.6	7	4.8	-0.021 ^b^	0.775
	≥ 15	82.3	59.2	30	66.7	13	28.9	2	4.4		
Teamwork	Acceptable (≥ 3)	80.1	50.9	6	18.8	22	68.8	4	12.5	-0.531 ^c^	< 0.001
	Unacceptable(< 3)	146.2	52.0	109	69.0	44	27.8	5	3.2		

a: Mann-Whitney test b: Pearson correlation coefficient c: Spearman’s correlation coefficient d: Kruskal Wallis

## Discussion

The present study set out to assess the correlation between teamwork and moral distress among female nurses in NICUs across Khorramabad and Semnan, Iran, in 2023. Findings revealed a noteworthy inverse relationship between teamwork and moral distress: as teamwork increased, nurses experienced lower levels of moral distress. This underscores the protective role cohesive teamwork can play in mitigating stress in high-pressure environments like the NICU. Previous research indicates that environmental and structural aspects within the NICU, such as inconsistent care teams, communication challenges, and staff shortages, contribute to moral distress, primarily due to nurses’ perceptions that the care delivered falls short of standards [42]. This research pioneers the direct correlation of teamwork with moral distress, setting a foundation for future studies to explore this relationship further.

Comparing the data on teamwork across different studies reveals varying results. For example, Profit et al. (2017) found that better teamwork was linked to lower rates of hospital-acquired infections in NICUs in California, USA [43]. Hosseini et al. reported a significant positive correlation between teamwork and nurses’ job motivation [44], while Pourmovahed et al. (2022) and Yaghoubi et al. (2019) found that better teamwork led to reduced missed nursing care in various healthcare departments [45, 46]. Additionally, a study by Nobahar et al. (2023) indicated an inverse relationship between teamwork, moral sensitivity, and missed nursing care [47].

The results of this study showed that the mean ± standard deviation of overall teamwork was 3.73 ± 0.78 out of 5. The average of all teamwork components was more than three. In other words, 83.2% of nurses had acceptable teamwork (≤ 3). In addition, more than 76% of nurses were acceptable in all components of teamwork. A study by Profit et al. (2017), in 44 NICUs in California, USA, showed that an average of 66% of participants reported teamwork at a good level [43]. The results of the study by Flin et al. (2006), among nurses and surgeons in Scotland show a positive attitude toward behaviors related to teamwork [48]. In the cross-sectional study of Nobahar et al. (2023), the mean ± standard deviation of the overall teamwork was 3.47 ± 0.69, which is in line with the results of the present study, although the aforementioned study was conducted on nurses in critical care units in two cities. Semnan, Shahroud, and not in NICUs [47]. Also, in the study of Yaghoubi et al. (2019), among the employees of the intensive care unit in Tehran, teamwork scored 3.67 out of 5, which indicates a moderate to high level of teamwork [46], which is similar to the results of the present study. In Hosseini et al.‘s study (2017), among the nurses of different departments in Kerman hospitals using the Hoegl and Gemuenden tool, the overall teamwork score was average and below average [44], which is inconsistent with the results of the present study. It seems that the cause of this difference, in addition to the difference in the study tool, there are other reasons, such as the studied department because Hosseini et al. research (2017) was conducted in different departments of the same hospital and not in NICUs, in other words, Teamwork, in different care units, is significantly different from each other [44].

In this study, the communication dimension scored the highest mean, reinforcing the notion that effective communication is pivotal in the NICU environment. This aligns with Kilner et al.‘s (2010) review asserting that proper communication within hospital teams, especially in emergencies, not only leads to better patient outcomes but also minimizes errors and fosters staff satisfaction [49]. The consistency of this finding with that of Hosseini et al. (2017) bolsters the argument for prioritizing communication in team development initiatives [44]. However, the discrepancy with Hekmat et al. (2015) findings, where communication scored lowest, can potentially be attributed to the different demographic profiles, as Hekmat’s study was not limited to nurses but included all members of the clinical committees in hospitals [50]. Despite the same tools, it seems that the reason for this difference in the results is related to the differences in the research community because the current research was conducted on NICU nurses, and Hekmat’s research was conducted on all members of the clinical committees in hospitals and not only on the group of nurses.

The present study also found that situational assessment had the lowest mean score. This mirrors the findings of Nobahar et al. [47] yet contrasts with the study of Weaver et al. (2017), where this dimension was rated highly among emergency room nurses and doctors in Texas [51]. The divergence suggests that factors like organizational and cultural context, as well as the specific nature of the emergency setting, may significantly influence how situational assessment is perceived and practiced.

Moral distress levels in NICU nurses were reported to be low on average, with a majority of the nurses falling within the low-distress category. This observation is consistent with findings from Carletto et al. (2022) in Italian NICUs among doctors, nurses, and physiotherapists [26]. and Khoshkbari et al. (2022) reported a low level of moral distress in the nurses of critical care units in Zanjan [52] and Zarei Nodei in nurses of intensive care units in Qazvin province [53], which is in line with the results of the present study. However, there is a considerable contrast with studies from Tajalli et al. (2021) in Tehran [5] and Tahmasebi et al. (2022) in the nurses of pediatric intensive care units in Tehran [54], Barkhordari et al. (2020), in the nurses of hospitals affiliated to Shahid Sadoughi University of Medical Sciences in Yazd [55], reported moderate moral distress. Further variation is seen in studies like Larson et al. (2017), which identified high moral distress among healthcare workers in Canadian PICU and NICU departments [56]. These different levels of moral distress may, as suggested, reflect variations in social and cultural backgrounds, differences in study tools, or organizational environments where the studies were conducted. It is suggested that the experience of moral distress is dynamic, potentially fluctuating according to external conditions and the internal coping mechanisms of individuals [20].

The findings from this multiple analysis indicate that among the demographic factors, only marital status and number of children have a significant relationship with moral distress.

More specifically, the study reports higher levels of moral distress among married nurses compared to their single counterparts, an observation that is not entirely consistent with previous research by Carletto et al. (2022), Tajalli et al. (2021), and Shamsaei et al. (2020), which found no statistical link between marital status and moral distress [5, 26, 57]. However, it aligns with the findings of Barkhordari et al. (2020) and Behbodi et al. (2018), who identified a significant relationship between moral distress and marital status [55, 58], suggesting married individuals may face greater psychological and mental strain due to dual responsibilities at work, patients and caring for them.

Interestingly, this study suggests that nurses with children experience lower levels of moral distress than their childless counterparts, which contrasts with the findings from Mohamadi et al. (2019) and Tahmasebi et al. (2022), who either found no relationship or reported higher moral distress in nurses with one child, respectively [54, 59], which is contrary to the results of the present study. In addition, in the study of Tahmasebi et al. (2022) in PICU nurses in Tehran, the level of moral distress in nurses with one child was higher than in nurses without children [54], which is contrary to the results of the present study.

Probably, the experience of having a child and being in situations where challenges in personal life have been solved in the past have helped NICU nurses cope with moral distress. However, the effect of having a child on the level of moral distress in NICU nurses remains unknown and has been investigated only in a few studies in the ICU and PICU departments, and conflicting results have been obtained. Therefore, more studies in this field seem necessary.

## Conclusion

Overall, teamwork within the NICUs was gauged as satisfactory, and the general level of moral distress was considered low. The study infers that having children seems to buffer NICU nurses against moral distress and that enhancing teamwork could further decrease its prevalence. Nursing managers can play a crucial role in reducing moral distress in NICUs by implementing changes in care methods and fostering a team-based approach, ultimately enhancing the quality of nursing care for neonates. Emphasizing teamwork among nurses and addressing moral distress is essential to the effective performance of NICU nurses. A more positive moral atmosphere in the NICU is associated with greater teamwork among nurses, which in turn leads to lower levels of moral distress. The nursing profession needs to recognize the importance of moral distress and identify the factors that contribute to its occurrence and exacerbation. Developing effective strategies to address this issue, particularly in critical care units like the NICU where neonates are in critical condition, is imperative. The findings of this study are therefore of significant importance for improving nursing care in the NICU. The teamwork of nurses in the NICU has been shown to significantly reduce moral distress. Additionally, a positive moral atmosphere within the NICU can enhance nurse cooperation. Professional collaboration among nurses not only helps achieve care goals but also shortens the length of stay for neonates in the NICU and improves end-of-life care. Furthermore, it can mitigate the effects of moral distress, addressing challenges such as nurse turnover, job burnout, and both physical and mental health issues among nurses. Therefore, it is suggested that future research examines the inter-professionalism and moral distress.

### Limitations

The scope of this study was restricted to NICU nurses, excluding other healthcare professionals like physicians, physiotherapists, radiologists, and occupational therapists, who also play a critical role in patient care within the NICU context. Although it was tried that the participants were calm when completing the questionnaires and the environment of the NICU was calm, it was not possible to control the mental states of the participants by the researchers. Furthermore, the data collection relied on self-reported questionnaires, which might introduce subjective bias, potentially skewing the results. The cross-sectional design of the study also limits the understanding of causal relationships.

## Data Availability

The datasets generated and analyzed during the current study are not publicly available due to privacy protection and ethical considerations but are available from the corresponding author upon reasonable request.

## Abbreviations

NICU: Neonatal Intensive Care Unit
ICU: Intensive Care Unit
T-TPQ: Team STEPPS Team Perception Questionnaire
MDS-R: Moral Distress Scale-Revised
SD: Standard Deviation
